# Peering into the Kaleidoscope of Cyclodextrins

**DOI:** 10.3390/biom11010121

**Published:** 2021-01-19

**Authors:** Susana Santos Braga

**Affiliations:** LAQV-REQUIMTE, Chemistry Department, University of Aveiro, 3810-193 Aveiro, Portugal; sbraga@ua.pt; Tel.: +351-234370200

Cyclodextrins (CDs) are known to us for 130 years, yet they remain ever as new and as fascinating as in their early years, when Villiers marveled at the unexpected growth of “beautiful radiated crystals” in the alcoholic media of his experiments on bacterial fermentation of starches, or when Freudenberg struggled to solve the puzzle of their unusual shape and structure [1]. In the present day, cyclodextrins have grown into a large and sophisticated family of compounds owing to the skilled research teams that have developed a myriad of derivatives appropriate for the most diverse applications.

This Special Issue of *Biomolecules* on “Perspectives of Cyclodextrins” offers a glimpse into a kaleidoscopic collection of cyclodextrins, bulking together in different shapes and patterns as they form supramolecular adducts with various guest compounds, expanding into a plethora of different derivatives and spanning a rainbow of applications from medicine and pharmaceutics to food/nutraceutics and polymers.

## Fundamental Research: The Water Ballet and the Cyclodextrin Mudra

Cyclodextrins are included in many water-based pharmaceutical and cosmetic emulsions as solubilizers and stabilizers, in tandem with surfactants and a variety of other ingredients. Still, knowledge on the interactions between CDs and these ingredients is limited. Less explored even is the effect of such interactions on the properties and distribution of water molecules—the main component of said emulsions. In the work of Garrido et al., the behavior of water molecules around (α-CD)_2_·SDS (SDS denotes sodium dodecyl sulfate) was simulated, showing fast dynamics of hydrogen bonding [2]. Water molecules are distributed around the units of the complex in symmetric choreographies, as if the water molecules themselves were synchronized swimmers: they form a hexagon around the apolar water-exclusion area of the mid-transversal section of the α-CD ring, and, at the rims, hexasymmetric, flowerlike contact areas with the host.

The topography of inclusion complexes with methylated cyclodextrins is rather less studied than that of their native counterparts. Lee et al. described adducts formed between a family of hydroxycinnamic acid derivatives and hexakis (2,3,6-tri-O-methyl)-α-CD (TRIMEA), heptakis (2,6-di-O-methyl)-β-CD (DIMEB), and heptakis (2,3,6-tri-O-methyl)-β-CD (TRIMEB), finding not only inclusion complexes with the typical “cage” and “channel” packing modes but also a rare example of a class of adducts called non-inclusion complexes [3]. Formed between DIMEB and ferulic acid, this adduct features guest molecules revolving around columns having merely host molecules, with one molecule of DIMEB partially included into the next—the same as one hand resting on the concavity of the other in the meditation mudra *dhyana*.

## Unveiling More Properties and Applications of Cyclodextrins

Cyclodextrins find various applications in pharmaceutics, from being simple solubilizers to sustained-release agents. Now, self-assembled nanoparticles formed by a new family of o-lipidyl cyclodextrins are opening the way to controlled delivery systems with high loading capacity [4]. These CDs are obtained using sustainable mechanochemical procedures and biocompatible with intestinal epithelial cells, thus promising a bright and green future in pharmaceutics. Another derivative, (2-hydroxy)propyl)-β-CD (HPBCD), is already approved for human use, serving as a solubilizer for various drugs, as an orphan medicine for Niemann–Pick type C, a rare brain disease, and being under evaluation for focal segmental glomerulosclerosis, a kidney disease [1]. A new biodistribution study uses a fluorescently labeled HPBCD analogue to show that only 30 min after being injected into mice, and besides the known kidney targeting, this cyclodextrin also builds up in the lung, liver, and brain [5], thus contributing to understanding the pharmacokinetics of HPBCD and potentially opening the way for other routes of delivery.

Cyclodextrins are also slowly making their way into nutraceutics, a fast-growing and exciting field that uses the beneficial properties of natural compounds and plant extracts to produce superfoods via fortification processes. A good example is found in the work of Pais et al., describing the extraction of 6-, 8-, and 10-gingerols from fresh ginger rhizomes and their inclusion into γ-CD to obtain an inclusion complex that not only retains the antioxidant activity of gingerols and but also affords, once incorporated into yogurt, an antioxidant-rich food with a mild spicy flavor [6]. The strong pungency of gingerols is masked by γ-CD inclusion. In another work, HPBCD inclusion is used to sharply increase the antioxidant power of idebenone, an analogue of coenzyme Q10 [7]. Cyclodextrins can also help keep food safe from contamination with mycotoxins by contributing to their detection and entrapment, as reported by Faisal et al. [8] and Fliszár-Nyúl et al. [9].

In polymer science, cyclodextrins can be used to include polymeric chains and template them into ordered and aligned forms that, after removal of the cyclodextrin, give rise to variants of the polymer materials with unique morphologies and improved thermal and mechanical properties [10].
